# Investigation of the Effect of Ultraviolet Light Treatment on *Enterobacterales* and *Salmonella* spp. in Raw Chicken Products

**DOI:** 10.1002/vms3.70577

**Published:** 2025-09-04

**Authors:** Nuray Gamze Yörük

**Affiliations:** ^1^ Department of Food Hygiene and Technology, Faculty of Veterinary Medicine University of Dokuz Eylül İzmir Türkiye

**Keywords:** *Enterobacterales*, public health, raw chicken parts, *Salmonella* spp, ultraviolet light (UV‐C)

## Abstract

The aim of this study was to evaluate the effect of UV‐C light (254‐nm wavelength, minimum intensity 0.573 mW/cm^2^) on the presence of *Salmonella* spp. and the counts of *Enterobacterales* in various raw chicken parts. *Salmonella* spp. was initially detected in 16 out of 20 samples. Following UV‐C treatment, the prevalence of *Salmonella* spp. positive samples progressively decreased with 13 positive samples remaining after 3 h and further reduction to seven samples after 6 h of exposure. Complete inactivation of *Salmonella* spp. was observed following after 9, 12, and 24 h of UV‐C application, ensuring microbiological safety in accordance with food safety standards. Additionally, *Enterobacterales* counts also showed significant reductions, particularly between 6 and 12 h of UV‐C treatment. These findings highlight UV‐C light as a promising intervention for mitigating foodborne pathogens in poultry products, with potential for practical applications in food safety systems. Further research with a larger number of samples is recommended to optimize UV‐C treatment for controlling foodborne pathogens in poultry food products.

## Graphical abstract

This study evaluated the effect of UV‐C light (45‐cm length, 254‐nm wavelength, minimum intensity 0.573 mW/cm^2^) for 3, 6, 9, 12, and 24 h on microbial contamination in raw chicken products. UV‐C treatment significantly reduced *Salmonella* spp. and *Enterobacterales* levels, with the most effective reduction observed after 6 h of exposure. These findings highlight UV‐C light as a promising intervention for mitigating foodborne pathogens in poultry products, with potential for practical applications in food safety systems.

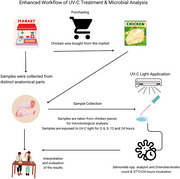

## 1 Introduction

Poultry products, particularly meat and eggs, are integral to diverse culinary traditions worldwide and are acknowledged as a highly efficient protein source (Mottet and Tempio 2024; Wessels et al. 2021). However, in recent years, foodborne illnesses have posed a significant public health challenge. The World Health Organization (WHO) estimates that globally foodborne diseases affect ∼600 million people annually, leading to 420,000 deaths with a considerable proportion attributed to contaminated poultry products (WHO 2022). In the United States, federal authorities estimate that annually ∼48 million individuals experience food‐related illnesses, resulting in around 128,000 hospitalizations and 3000 fatalities (CDC 2019). Similarly, research from the United States has demonstrated that *Salmonella* contamination in poultry contributes to a substantial burden of foodborne disease, with an estimated 1.35 million infections annually (CDC 2021).

The situation is also concerning in other regions, with Central Asia and Europe reporting over 23 million cases of foodborne illnesses yearly, leading to about 5000 deaths. Australian officials estimate that their country faces ∼5.4 million cases of food‐related illnesses annually (Hamaideh et al. 2024). The Foodborne Diseases Active Surveillance Network found that in 10 US sites in 2018, *Salmonella*, *Escherichia*, and *Listeria* caused 126, 2925, and 9084 cases, respectively (Kumar et al. 2020).

*Enterobacterales* family bacteria contaminate animal feed through raw materials, causing foodborne illnesses (Maciorowski et al. 2004). Particularly, *Salmonella* is one of the most common pathogens in poultry and eggs (Antunes et al. 2016; Chai et al. 2017; Majowicz et al. 2010) and spreads through the consumption of infected animal products (Adetunji et al. 2022).

Transmission of extended spectrum beta‐lactamase (ESBL)‐producing *Escherichia coli* from animals to humans may occur readily through the food production chain, especially in broiler chicken farms (Lazarus et al. 2015; Vitas et al. 2018). In light of these growing challenges, non‐chemical interventions have gained increased attention. Among them, UV‐C light—a short‐wavelength ultraviolet light known for its germicidal properties—has emerged as a promising method to reduce microbial loads, including drug‐resistant pathogens, on food surfaces (Pihen et al. 2023; Singh et al. 2021; Delorme et al. (2020).

UV‐C light emerges as an effective method for reducing *Salmonella* and *Enterobacterales* bacteria in different raw chicken products to ensure food safety. This study investigates the effects of UV‐C light application at different durations on these bacteria.

## 2 Material and Methods

### 2.1 Sample Collection

A total of 20 raw chicken samples were collected from local markets in the Kocaeli province, Türkiye, using a purposive sampling method to represent various anatomical parts, including liver‐heart (*n* = 3), grilled skinless chicken leg (*n* = 1), chicken drumstick (*n* = 3), grilled chicken wing (*n* = 1), skin‐on chicken leg (*n* = 2), chicken wing (*n* = 4), chicken thin‐leg (*n* = 2), chicken roll (*n* = 3), and chicken fillet (*n* = 1). A purposive sampling was employed to ensure diversity in sample types and target commonly consumed poultry parts. This anatomical diversity enabled examination of microbial responses across distinct tissue types and surface properties. Given the labour‐intensive nature of microbiological analysis—including serial dilutions, aseptic plating, and incubation protocols—the number of samples was determined to ensure procedural precision and reproducibility while maintaining sufficient anatomical diversity for comparative assessment. For quantitative analysis, decimal dilutions were prepared using 9 mL of sterile buffered peptone water (BPW) (LAB M–LAB204, UK), and subsequent inoculations were performed under aseptic conditions.

### 2.2 *Salmonella* spp. and *Enterobacterales* Analyses

#### 2.2.1 Presence of *Salmonella* spp

The samples were cut into small pieces with a sterile scalpel under aseptic conditions, and 25 g of each sample was aseptically weighed into sterile disposable TEMPO stomacher bags (BioMérieux). About 225 mL of sterile BPW (LAB M‐ LAB204) was added, and a homogeneous mixture was obtained in a stomacher. Subsequently, the samples in BPW were incubated at 37°C for 18 ± 2 h for pre‐enrichment (ISO 6579‐1: 2017). After incubation, 0.1 mL of the pre‐enriched sample was inoculated into 10 mL of Rappaport‐Vassiliadis (RVS‐LAB M‐ LAB086) and 1 mL of the sample into 9 mL of Muller‐Kauffmann Tetrathionate‐Novobiocin broths (LAB M‐ LAB202). RVS broths were incubated at 41.5°C for 24 ± 2 h, and Muller‐Kauffmann Tetrathionate‐Novobiocin broths were incubated at 37°C for 24 ± 2 h. For isolation after selective enrichment, Xylose Lysine Deoxycholate (XLD‐LAB M‐ LAB032) agar was plated with a disposable sterile core according to the smear plate method, and the plates were incubated at 37°C for 18–24 h. Black bead‐like colonies were evaluated as suspicious. An oxidase test was performed, and suspected strains were identified using the API 20E Biochemical test (BioMérieux‐REF 20100). The results were serologically confirmed using Vi (Microgen‐BTA111), O (Microgen‐BTA002), and H (Microgen‐BTA121) antigens.

#### 2.2.2 **E**numeration of *Enterobacterales*

For the enumeration of the *Enterobacterales* counts, 1 mL of the respective samples was plated on to Violet Red Bile Glucose Agar (VRBGA‐LAB M‐ LAB088). Upon cooling, another layer of VRBGA was poured on the media left to solidify and after the media were solidify, all the inoculated petri dishes were left to incubate at 37°C for 24 h. At the end of incubation, the violet purple colonies in the petri plates were regarded as positive. For confirmation, suspected colonies were grown in 10 mL Glucose of Medium (Liofilchem‐REF 610388) at 37°C for 24 h. The samples were evaluated as glucose‐positive upon a change in their color to yellow (ISO 21528‐ 2:2017).

After homogenization, 1 mL of the mixture was transferred into sterile disposable Petri dishes. Violet Red Bile Glucose Agar (VRBGA‐LAB M‐ LAB088) was added to the samples in sterile petri dishes so that the medium was 10–12 cm. Once the medium had solidified, an additional layer of VRBGA agar was poured over it and allowed to solidify again. Then, all of the inoculated petri dishes were left to incubate at 37°C for 24 h. At the end of incubation, the violet purple colonies in the petri dishes were counted. For confirmation, 10 mL Glucose of Medium (Liofilchem‐REF 610388) in Suspect Colony vertical drawing was made from suspected *Enterobacterales* colonies and incubated obliquely at 37°C for 24 h. Growths were formed in Glucose of Medium (Liofilchem‐REF 610388) in which *Enterobacterales* were detected. Glucose‐positive reactions were identified by a colour shift from red to yellow in the glucose‐containing tubes (ISO 21528‐ 2:2017).

#### 2.2.3 UV‐C Light Application

**FIGURE 1:**
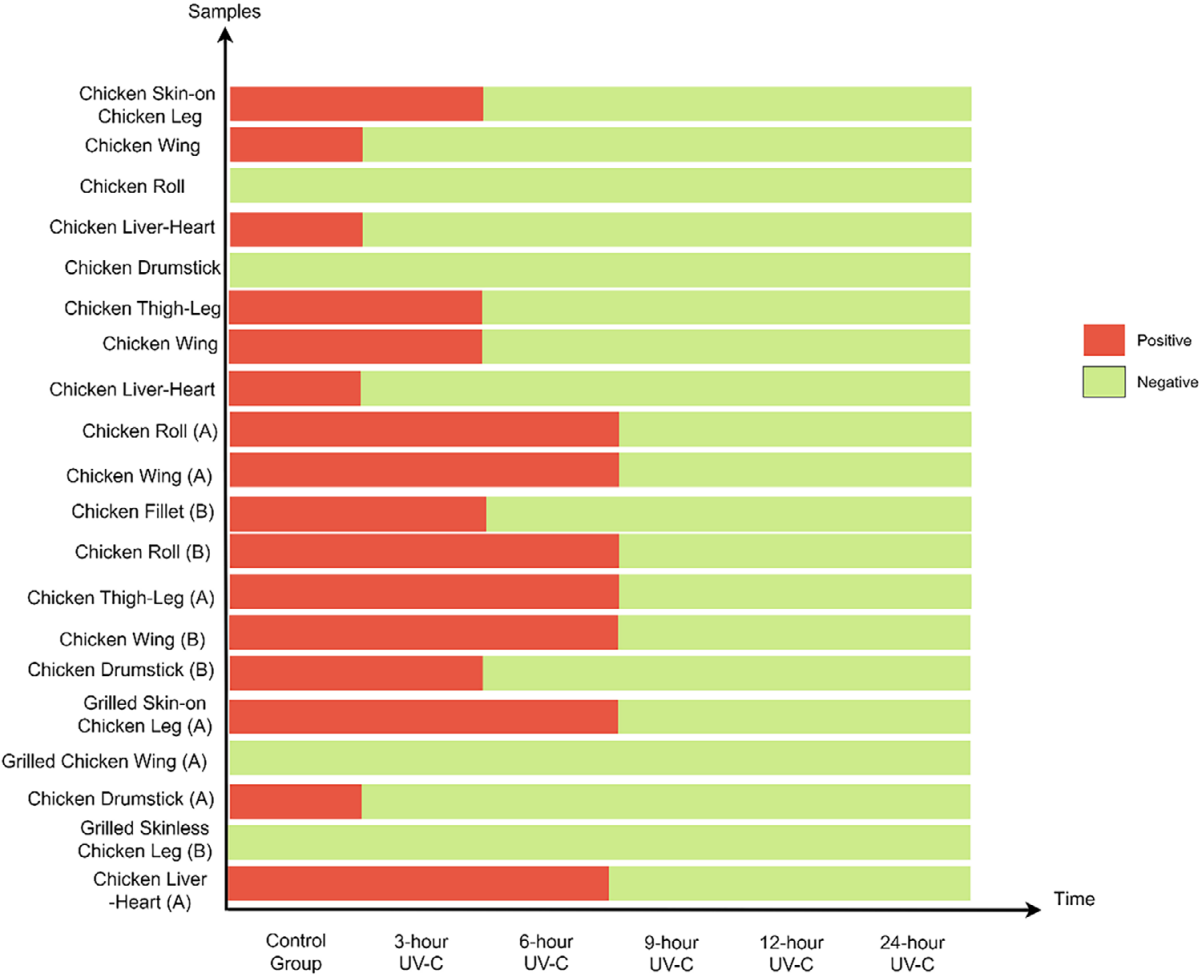
Bar graphic representation of biochemical confirmation test results (*Salmonella* spp.). Control group: Non‐UV‐C treated group. Positive: It shows poultry products that are still positive after UV‐C treatment at baseline and for certain periods of time. Negative: It shows poultry products that are negative with UV‐C treatment at baseline and for certain periods of time.

This study's methodology involved analyzing 20 chicken parts for *Salmonella* spp. and *Enterobacterales* counts using ISO standards. Investigation of the effect of UV‐C on *Salmonella* spp. and *Enterobacterales* counts on various anatomical parts in chicken was carried out using a bench scale UV‐C light system. The system operated at a wavelength of 254 nm wavelength and 15 watts. The products were exposed to a UV‐C light (45‐cm length) for 3, 6, 9, 12, and 24 h (Figure 1). The UV‐C application was conducted at 12°C, which is in compliance with the production area temperature, with the lamp placed at a distance of 50 cm from the samples. The UV‐C light had a minimum irradiance of 0.573 mW/cm^2^. The research focused solely on the microbiological effects of UV‐C on chicken parts, maintaining consistent temperature and distance parameters throughout the experiment.

### 2.3 Statistical Analyses

Data normality was tested with the Shapiro–Wilk test, and non‐parametric tests were employed. The Friedman test was used to compare differences for repeated measures of UV‐C exposure durations, and the Wilcoxon signed‐rank test was applied for pairwise comparisons.

## 3 Results

In the study, application of UV‐C treatment resulted in a substantial reduction in microbial contamination. *Salmonella* spp. was initially detected in 80% (16/20) of the samples. Following 3 h of UV‐C exposure, the prevalence of *Salmonella* spp. positive samples declined to 65% (13/20), further reducing to 35% (7/20) after 6 h. Complete inactivation of *Salmonella* spp. was achieved after 9, 12, and 24 h of exposure (Figure 1).

The Friedman test indicated significant differences between the control and UV‐C‐treated groups for *Salmonella* spp. (*χ* ^2^ = 47.808, *p* < 0.05). Salmonella levels were significantly reduced after 3 h of UV‐C treatment (Table 1).

**TABLE 1:** Evaluation of *Salmonella* spp. levels according to UV‐C application periods.

	$\bar{x}$	Std.	Mean rank	*χ* ^2^	*p*
Control group	2.28	0.24	5.65	47.808	<0.001
3‐h UV‐C	2.23	0.22	5.20		
6‐h UV‐C	2.00	0.26	4.15		
9‐h UV‐C	1.02	0.22	3.00		
12‐h UV‐C	0.64	0.26	2.00		
24‐h UV‐C	<0.001	<0.001	1.00		

Similarly, *Enterobacterales* counts exhibited statistically significant reductions over time, with notable decreases observed at 6 h (*p* = 0.01), 9 h (*p* = 0.00), 12 h (*p* = 0.01), and 24 h (*p < 0.001*) when compared to the control group (Table 2). These findings underscore the efficacy of prolonged UV‐C treatment in mitigating bacterial contamination in raw poultry.

**TABLE 2:** Wilcoxon signed‐rank test results of the effect of UV‐C applications on *Enterobacterales* levels.

	*Z*	*p*
3‐h UV‐C‐control group	−1.829	0.07
6‐h UV‐C‐control group	−2.797	0.01
9‐h UV‐C‐control group	−3.823	<0.001
12‐h UV‐C‐control group	−2.803	0.01
24‐h UV‐C‐control group	−3.825	<0.001
6‐h UV‐C‐3‐h UV‐C	−3.92	<0.001
9‐h UV‐C‐3‐h UV‐C	−3.92	<0.001
12‐h UV‐C‐3‐h UV‐C	−2.803	0.01
24‐h UV‐C‐3‐h UV‐C	−3.92	<0.001
9‐h UV‐C‐6‐h UV‐C	−3.92	<0.001
12‐h UV‐C‐6‐h UV‐C	−2.803	0.01
24‐h UV‐C‐6‐h UV‐C	−3.921	<0.001
12‐h UV‐C‐9‐h UV‐C	−2.803	0.01
24‐h UV‐C‐9‐h UV‐C	−3.826	<0.00
24‐h UV‐C‐12‐h UV‐C	−2.809	0.01

The results of the Wilcoxon signed‐rank test assessed the relationships between UV‐C applications at different time intervals and their control groups, as well as comparisons between different time intervals for *Enterobacterales*. The initial comparison between the 3‐h UV‐C group and the control showed no significant difference (*Z* = −1.829, *p* = 0.07). However, in comparison with the 6‐h (*Z* = −2.797, *p* = 0.01), 9‐h (*Z* = −3.823, *p < 0.001*), 12‐h (*Z* = −2.803, *p* = 0.01), and 24‐h (*Z* = −3.825, p < 0.001*p*) UV‐C control groups, statistically significant differences were observed, indicating the effectiveness of UV‐C applications compared to their respective control groups. Additionally, comparisons between different time intervals of UV‐C applications also generally revealed significant differences. Specifically, in the comparison between 3 h and 6 h (*Z* = −3.92, *p < 0.001*), and between 9 h and 6 h (*Z* = −3.92, *p < 0.001*), a significant impact of UV‐C treatment on *Enterobacterales* levels was detected (Table 2).

The amount of difference between the control group and 6‐h periods is significant for chicken drumsticks and chicken fillet samples, with (*p* < 0.05). Drumsticks and fillets differ from other samples by becoming sterile after 6 h.

Regarding the changes between the 3‐h and 6‐h periods, a significant increase is observed in skinless grilled chicken thighs. On the other hand, there is a significant decrease in chicken drumsticks and chicken fillet samples, all with (*p* < 0.05).

When comparing the logarithmic mean changes among the groups at 0, 3, 6, 9, 12, and 24 h, significant differences were found between the two repeated measurement averages at these time points (*p* < 0.001). Specifically, significant differences were observed in the samples of chicken liver‐heart, chicken drumsticks, chicken drumsticks with skin, chicken grilled wings, chicken drumsticks with skin, and chicken drumsticks with skin.

Additionally, the repeated measurement averages between 0, 3, 6, 9, and 12 h in the samples of chicken wrap (9th and 12th) showed a significant difference (*p* < 0.05).

## 4 Discussion

*Salmonella* contamination of poultry meat remains a major public health problem on a global scale. Majowicz et al. (2010) estimated that foodborne *Salmonella* gastroenteritis causes ∼93.8 million cases and 155,000 deaths annually worldwide. Poultry products are among the major sources of these infections. Antunes et al. (2016) reported that the widespread use of antibiotics in poultry production in Brazil contributed to the development of significant levels of antimicrobial resistance in *Salmonella* strains. Similarly, Tagar and Qambrani (2023) reported that 97.3% of *Salmonella* spp. were found in retail chicken meat in Pakistan and that these isolates exhibited multidrug resistance. Lunara et al. (2023) detected *Salmonella* spp. at a rate of 46.1% in chilled chicken meat offered for retail sale in Brazil and reported that these isolates carried resistance genes such as *sul2, blaCTX*, and *tetB*. These findings emphasize the prevalence of *Salmonella* contamination in poultry products and the problem of antimicrobial resistance. Given its established role as a major foodborne pathogen causing severe gastrointestinal infections, particularly among vulnerable populations such as immunocompromised individuals, children, and the elderly, the complete elimination of *Salmonella* through UV‐C treatment observed in this study is notably significant (CDC 2021). The successful eradication of *Salmonella* spp. after prolonged UV‐C exposure underscores its considerable potential as an effective intervention for enhancing food safety. Similar observations have been documented by previous studies, where prolonged UV‐C application effectively decreased bacterial loads without compromising meat quality, further supporting its utility in food processing environments (Byun et al. 2022; Vatansever et al. 2013).

In addition to its efficacy against *Salmonella*, UV‐C treatment demonstrated significant effectiveness in reducing *Enterobacterales* contamination. The present study highlighted a clear, time‐dependent decrease in *Enterobacterales* levels starting at 6 h of exposure, with continued reductions observed at longer exposure times (9, 12, and 24 h). Notably, chicken drumstick and fillet samples exhibited a marked reduction in *Enterobacterales* counts, achieving complete sterility after 6 h of UV‐C treatment. Conversely, some poultry products, such as skinless grilled chicken thighs, showed variable microbial responses. These variations suggest that specific product characteristics—including surface properties, initial contamination levels, and intrinsic factors such as tissue structure—may impact the effectiveness of UV‐C irradiation. These findings align with earlier research by Wang et al. (2021), which reported an ∼1.90–2.25 log CFU/cm^2^ reduction in *Enterobacterales* populations following UV‐C LED treatment on chicken breast samples. Similarly, Byun et al. (2022) demonstrated enhanced microbial reduction when combining UV‐C exposure with additional antimicrobial agents, emphasizing the potential benefits of multi‐hurdle food safety strategies. Therefore, the use of UV‐C irradiation appears highly promising for controlling *Enterobacterales* contamination, particularly with treatment durations exceeding 6 h. Future studies should further optimize UV‐C parameters, including exposure duration, intensity, and irradiation distance, to maximize microbial inactivation across various poultry products in industrial processing contexts.

Several studies supporting our findings have emphasized the effectiveness of UV‐C irradiation in reducing microbial loads in meat products. For example, Keklik et al. (2012) showed a two‐log reduction of *Salmonella Typhimurium* in boneless chicken breast samples after short‐term exposure (5–60 s) to UV‐C at 260 nm. Yeh et al. (2018) investigated the combined use of UV‐C and bacteriophage on minced meat and reported a one‐log reduction with UV‐C alone and enhanced effects (up to two‐log reduction) when combined with lactic acid and peroxyacetic acid. Calle et al. (2021) showed a direct relationship between increasing irradiation parameters and bacterial reduction, emphasizing the importance of light intensity and exposure time. Similarly, McLeod et al. (2018) observed microbial reductions ranging from 0.9 to 3.0 log CFU/cm^2^ in chicken fillets, reinforcing the practical potential of UV‐C treatment to improve poultry safety.

Geographical and seasonal variations in *Salmonella* prevalence also underline the complexity of controlling this pathogen in poultry products. Li et al. (2020) reported higher *Salmonella* prevalence in raw chicken during spring and summer in Shaanxi Province, China, with dominant serotypes demonstrating multi‐drug resistance. Similarly, Tagar and Qambrani (2023) identified extensive *Salmonella* contamination (over 90%) on meat‐cutting surfaces and meat products in Pakistan. Ishihara et al. (2020) also indicated seasonal peaks of *S. Schwarzengrund* contamination in broiler meat during spring and winter months in Japan. These findings emphasize the need for region‐specific strategies and targeted interventions such as UV‐C treatment to effectively manage microbial contamination in diverse settings.

Mechanistically, UV‐C irradiation at 254 nm disrupts microbial DNA/RNA, impairing cellular reproduction and essential functions, thereby preventing pathogen replication (Rastogi et al. 2010; Tchonkouang et al. 2023). Importantly, UV‐C treatment has not been associated with the formation of detectable mutagenic compounds, supporting its safe application for consumer food products (Vatansever et al. 2013). Consequently, UV‐C irradiation represents a robust, non‐chemical alternative for microbial control, offering significant potential to enhance food safety and public health globally.

### 4.1 Limitations of Study

Among the limitations of this study, firstly, there is the question of how the results obtained in the laboratory environment will be reflected in real‐world conditions. Although our experiments were conducted under controlled laboratory conditions, interactions between various environmental factors in real‐world settings could affect the outcomes.

## 5 Conclusion

UV‐C light shows promise for controlling *Salmonella* spp. and *Enterobacterales* in raw chicken products, effectively reducing overall microbial contamination. However, its effectiveness varies based on application time and product type. This “hurdle technology” is cost‐effective for food hygiene monitoring in underdeveloped nations due to low maintenance, installation, and operation costs. Further research is needed to understand UV‐C interactions with various parameters and microorganisms, optimizing treatment protocols for food safety applications.

## Author Contributions

The author made substantial contributions to the conception or design of the work, the acquisition, analysis, interpretation of data, the creation of new software used in the work (statistical analysis), wrote the main manuscript, prepared graphical abstract, figures and tables, reviewed and revised the manuscript.

## Ethics Statement

The present research was completed without any animal experimentation. This investigation adhered strictly to ethical guidelines established by both institutional and national research committees, in full alignment with the foundational principles set forth in the 1964 Helsinki Declaration, including all subsequent modifications and equivalent ethical frameworks.

## Conflicts of Interest

The author declares no conflicts of interest.

## Data Availability

The data supporting the findings of this study are available upon reasonable request from the corresponding author.
